# Study of the Hepatoprotective Activity of Methanolic Extract of *Feijoa sellowiana* Fruits Against MDMA using the Isolated Rat Liver Perfusion System

**Published:** 2013

**Authors:** Mohammad Karami, Sodabah Saeidnia, Anahita Nosrati

**Affiliations:** a *Department of Toxicopharmacology, School of Pharmacy, Mazandaran University of Medical Sciences, Sari, Iran. *; b *Medical Plants Research Center, Tehran University of Medical Sciences, Tehran, Iran. *; c *Pathology Department, School of Medicine, Mazandaran University of Medical Sciences, Sari, Iran. *

**Keywords:** *Feijoa sellowiana*, Liver perfusion, Kerbs-Henseleit buffer, MDMA, SGOT, SGPT, GSH

## Abstract

liver injury induced by viruses, chemicals and drugs can be protected by different medicinal plants. *Feijoa sellowiana *(Myrtaceae) is an evergreen bush native to southern areas of South America, as well as Iran where the fruits are very popular. Feijoa has shown a potent antimicrobial effect. Morever, the antioxidant activity of total Feijoa extract has also been reported. MDMA or ecstasy is a ring-substituted amphetamine derivative which has been abused as a widespread recreational drug by the young generation. Liver is a target organ for MDMA toxicity. In fact, this sense MDMA is metabolized by cytochromes P_450_2D, 2B and 3Aand reactive metabolites are readily oxidized to the corresponding o-qiuinones and reactive oxygen species (ROS).

This study investigated whether methanilic *Feijoa sellowiana *fruits can produce biochemical changes using the Isolated Rat Liver Perfusion (IRLP) system. The, the liver was perfused with different concentrations of the extract (10, 20, 40, 50,100 mg/kg), added to the buffer and perfused within 2 h. During the perfusion we tried to find out the antioxidant activity or liver protective effect of Feijoa, by determinining amino-transferases activities (SGOT and SGPT ) and glutathione reductase (GSH) level in comparison with the positive and negative controls. Subsequently, sections of liver tissue were examined for any histopathological changes. The results revealed that the activities of SGOT and SGPT were seriously decreased and GSH level was significantly increased by the Feijoa extract. Overall, necrosis in the liver parenchyma was decreased. These findings revealed that *Feijoa sellowiana *is an effective hepatoprotective plant.

## Introduction

*Feijoa sellowiana *(Myrtaceae) is an evergreen bush native to southern areas of South America, where it is widely distributed. Owing to its easy adaptability in subtropical regions, nowadays it is extensively cultivated in many countries (1), and also in Iran where the fruits are very popular. Although the chemical composition of Feijoa has been clearly reported (2-6), in-depth studies on its constituents have barely been carried out. Feijoa has shown potent antimicrobial and antifungal activities and a sensible activity against *H. pylori *(7-9). Anti-cancer activities of the total Feijoa extract have also been presented (10, 11). Moreover, antioxidant activities of an aqueous extract on oxidative burst of human whole blood phagocytes have been described (5, 11). Yet, little information is available on Feijoa antioxidative activity. In this study, we examined the antioxidant activity of the methanolic extract of *Feijoa sellowiana *against damage induced by MDMA in mouse liver, in order to understand the usefulness of this plant as a foodstuff as well as in medicine.

3, 4-methylenedioxymethamphetamine (MDMA or ecstasy) is a ring-substituted amphetamine derivative, wich was synthesized in 1912 by Merck chemical company and has attracted a great deal of media attention in recent years due to its widespread abuse as a recreational drug by the youth (12, 13). Clinical evidence has shown that the liver is a great target for MDMA toxicity. In fact, MDMA is metabolized by cytochromes P_450_2 D, 2B and 3A and reactive metabolites are readily oxidized to the corresponding o-qiuinones and in the long run formation of reactive oxygen species (ROS) (14,15). In the present survey we attempted to find out the hepatoprotective effect of *Feijoa sellowiana *accompanied by assessment of aminotransferase enzymes activities (SGOT and SGPT), Hepatic glutathione reductase (GSH) level and liver histopathology, compart to the positive and negative controls.

## Experimental

*Animals*

Male albino Wistar rats (6 to 8 weeks), weighing 200-250 g were used for all experiments. They were housed pretty individually in standard rat cages in a room with a relative humidity of 50±5% on a 12 h light- dark cycle at 22°C (22 ± 1 °C). Animals had ad libitum access to food and water. The animals were adapted to the condition for 7 days, prior to the beginning of the experiment .The experiment performed during the day time (08:00 AM-16:00 PM). Each animal was used once only. A research proposal was prepared according to the guidelines of the Committee for the Purpose of Control and Supervision of Experiments on Animals (CPCSEA). The Institutional Animal Ethical Committee (IAEC) of Mazandaran University of Medical Sciences approved the proposal.

*Plant*

*Feijoa *fruits were collected from Fajr citrus experimental institute in autumn 2010. 

Fruit peels were dried at room temperature and coarsely grounded before extraction. A known amount of each part was extracted at room temperature, using the by percolation method, with methanol and water; methanol/ water;(80:20, 400 mL × 3 times) as the extraction solvent. The resulting extract was concentrated in a rotary vacuum, until a crude solid extract was obtained. The extract was freeze-dried for complete solvent removal (16). The extract was dissolved in phosphate buffer (pH = 7.4) for pharmacological studies. The fruit peels aqueous extract and fruit peels methanolic extract (FM, 20.5%) were obtained, respectively. 

*Experimental design*

Rats were divided into five treatment groups and control groups. Each group contained four male rats, and whose livers were perfused by a single dose of 10,20,40,50 and 100 mg/kg of aqueous extract of aerial parts of *Feijoa sellowiana*, respectively (total = 7 groups). Control livers were perfused with the perfusion buffer. Following the preliminary study, a dose of 100 mg/kg was chosen for the remaining of the study in order to evaluate the hepatoprotective activity of Feijoa (17).

*Buffer*

**Figure 1:**
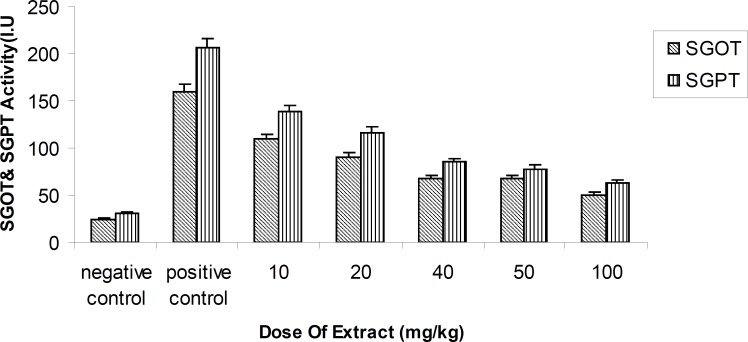
Activity of ALT (Alanine transferaes) and AST(Asparate transferaes) enzymes methanolic fruit extract of *Feijoa sellowiana *at differences concentrations . Values are presented as mean ± SEM (N = 5), ***p < 0.001 with respect to control, (ANOVA followed by Newman–Keuls multiple comparisons test).

Perfusion fluid was made of Kerbs-henseleit buffer. The perfusion medium consisted of 118.9mM NaCl, 4.76 mM KCl, 1.19mM KH_2_PO_4_, 2.55 mM CaCl_2_ and 24.8 mM NaHCO_3_, at 37°C. Glucose (1%W/V) was usually added (18), The perfusion medium was gassed continuously with carbogen (95%O_2_, 5%CO_2_) (Figure 1).

*Perfusion conditions and parameters of liver viability*

Temperature, perfusion pressure, flow rate and perfusion fluid pH were closely monitored during the perfusion time, particularly during the first 30 min of equilibration (18). These parameters were initially checked every 10 to 15 min and the experiment did not begin until they had reached constant and acceptable values. The temperature in the perfusion system was also set and maintained at 37°C. Perfusion pressure was not raised above 10-15 cm of water, with a flow rate of approximately 2 mL/min/g liver weight, in order to provide adequate oxygenation. The perfusion fluid pH was always set between 7.2 and 7.4, by adjusting the CO_2_ gas. As soon as the perfusion began, the liver displayed an even light-brown color. The liver was soft and kept moistened. Serum amino-transferases activities (ALT and AST) served as indicators of liver viability during perfusion, and was determined in samples of the perfusion medium. 

*Biochemical determinations*

The activities of Aspartate aminotransferase (AST) activity and Alanine aminotransferase(ALT) activity in sampels of perfusion medium were assayed using a commercial Kit of Zist chimie, (Tehran , Iran). Reduced glutathione (GSH) was estimated by the Ellman’s method (19). 

*Histological studies*

The liver was completely excised and all the extraneous tissue was meticulously stripped off. After that multiple samples were taken from each liver (mean diameter of 3 mm) and placed in 10% neutral buffered formalin. The liver was cut into small pieces, then sections were prepared and stained by Hematoxylin - Eosin and examined blindly for histopathological changes by an expert pathologist. 

*Surgery*

The rats were anesthetized with ether-Heparin (500 unit; IP) in order to prevent blood clotting prior to anesthesia (20). First of all an incision was made along the length of the abdomen to expose the liver. Next Sutures were placed loosely around the common bile duct, which was then was annulated with PE-10 tubing and secured. Sutures were placed loosely around the inferior vena cava, above and below the renal veins. The distal suture around the vena cava was tightened and an 18 g polyethylene catheter was inserted, and placed above the renal vein. Then, diaphragm was incised and the inferior vena cava ligated suprahepatically. At the end, following attachment of the perfusion tubing to cannulate, the liver was perfused *in-situ*, carefully, through the portal vein (20). 

*Data analysis*

Statistical analysis was performed using the SPSS for Windows (Ver.10, SPSS, Inc.Chicago, USA) software. All values were analyzed by the one-way analysis of variance (ANOVA) test and expressed as mean ± standard error of the mean (SEM), in 5 rats. 

Student-Newman-Keuls test was used to evaluate the significance of the obtained results. P < 0.05 was considered to be significant. 

## Results

*Activity changes in the serum amino-transferees enzymes*

**Figure 2:**
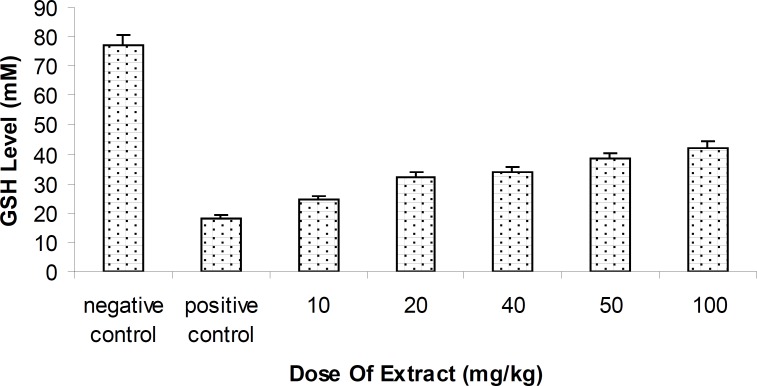
Level of Glutathione (GSH) methanolic fruit extract of *Feijoa sellowiana *at difference concentrations . Values are presented as mean ± SEM (N = 5) ***p < 0.001 with respect to control, (ANOVA followed by Newman–Keuls multiple comparisons test).

This study demonstrated that the methanolic extract of Feijoa fruits produced statistically significant results, such as a decrease in the activity of amino-transferases enzymes dose-dependently (p < 0.01) at 24^th^ h, in comparison with the positive control at a single dose of 10, 20, 40, 50 and 100 mg/kg (Figure 2). 

*Hepatic Glutathione reductase (GSH) level changes*

**Figure 3:**
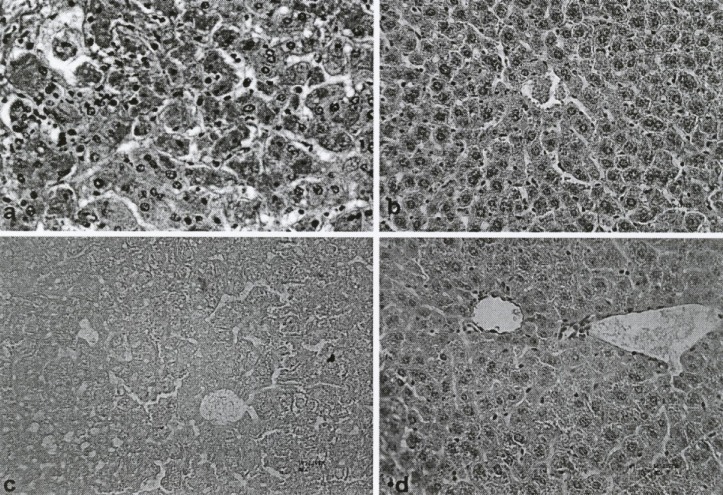
Photomicrograph of lobules from control groups and fruit extract of *Feijoa sellowiana *at differences concentration–treated liver. Staining of neagative Control shows that cytoplasm was acidophilic and surrounded by a bright basophilic nucleus (a). MDMA (5 mg/kg) or positive control showed limited changes in lobules of liver and hepatocellular necrosis, with infiltration of mononuclear cells and accumulation of necrotic Kupffer cells with Pyknotic nuclei (b). Histo-pathological changes of aqueous (for comparison) and methanolic extract of *Feijoa sellowiana *fruit at a single dose of 100 mg/kg, respectively (c and d).

Level of blood GSH of rats injected by methanolic extract of Feijoa fruit was relatively increased, compared with the positive control group . Antioxidant effect of Feijoa has been indicated to inhibit the hepatotoxic effect of MDMA (p < 0.05 ),.with respect to the control group (Figure 3).

*Light microscope observation*

Histopathological studies, using a light microscope, showed a meaningful decrease in hepatocellular damage, including necrosis and inflammatory cells infiltration, due to the use of the methanolic extract of Feijoa Fruit (Figure 4d), compared with the control group (Figure 4a). In addition, other histopathological parameters, such as the number of Kupffer and mononuclear cells, changed significantly with the methanolic extract of Feijoa fruit (Table 1). 

**Table 1:** Histo-pathological effects of the methanolic extract of *Feijoa sellowiana *fruit at different concentrations of 10, 20, 40, 50, 100 mg/kg.

**Histopathological ** **Parameters **	**Negative ** **Control (10mL/Kg) **	***Feijoa sellowiana*** **(mg/kg) ** **10 20 40 50 100 (mg/kg) **	**Positive ** **Control (5mg/kg) **
Kupffer cells	+	+3 +3 +3 +2^**^ +2^**^	+ 3^*^
Edematous cells	+	+1 +1 +1 +2^*^ +2^**^	+4^*^
Mononuclear cells	+	- +1 +1 +2 +2	+2
hemorrhage	_	+1 +1 +1 +2^*^ +3^**^	+4
Necrosis	_	- - +1 +2^*^ +3^**^	+ 5^** ^

-no effect, +1minor effect, +2medium effect, +3major effect, +4high effect, +5extra-high effect. ^*^p < 0.05, ^**^p < 0.01, significantly different from the control group using Fisher, exact test. Data are presented as means of three replicates

## Discussion

The liver has been identified as the most important target tissue for MDMA in mice (21). In order to elucidate the MDMA– induced hepatotoxicity, the effects of MDMA on transaminase enzyme activity and total glutathione mouse liver were determined. Moreover, the antioxidant effect antioxidant of Feijoa on MDMA–induced hepatotoxicity was assessed. 

In the present study, we pondered upon how the hepatotoxicity of MDMA can be prevented or reduced. The activity of aminotransdferase enzyme was increased significantly, which correlated well with decrease in glutathione (22). On the other hand, after the use of aqueous and methanolic extracts of Feijoa fruit as, an antioxidant , the hepatotoxic effect on enzymes was reduced by up to 40-50% compared to the control group. Glutathione deplection has been shown to correlate somehow with the lipid peroxidation in liver. It is believed that MDMA in ecstasy, is one of the primary toxic constituents. Other toxic constituents have also been identified, including MDA and DOM. In this study, MDMA induced formation of reactive oxygen species and an oxidative stress, resulting in lipid peroxidation (21, 23). Further studies, however, are needed to elucidate the exact mechanism by which MDMA induces hepatotoxicity. Moreover, MDMA was also shown to be an inhibitor of glutathione peroxidase, which catalyzes the destruction of H_2_O_2_ of lipid hydroperoxidase by reduced glutathione. Therefore, glutathione peroxidase inhibition can diminish GSH activity and actually resulted in an accelerated lipid peroxidation (24, 25). Antioxidants such as vitamin E and selenium have been proposed to prevent membrane damage of lipid peroxidation, not only through prohibiting glutathione peroxidation, but also by allowing hydrogen to be abstracted from their own structure (rather than from the allylic hydrogen of on unsaturated lipid ). Thus, interrupting the free radical chain reaction (26).Treatment with methanolic extract of *Feijoa sellowiana *fruit has been exhibited to importantly decrease the toxicity of MDMA (Table.1).This can come about through the mechanism mentioned above, as well as the fact that this extract may have good reductive capability for reducing Fe^+3^ to Fe^+2^ by donating an electron Fe^+2^ chelating activity and anti-lipid peroxidation activity (27). All in all, other extensive investigations on individual compounds, regarding their different *in-vivo *antioxidant activities, are required to be conducted.
